# Evaluation index system of education quality for nursing professional degree postgraduate using the analytic hierarchy process

**DOI:** 10.1097/MD.0000000000027771

**Published:** 2021-11-24

**Authors:** Wentong Wei, Jingying Liu, Yanhui Liu, Yannan Kang, Ruzhen Luo, Xiaohong Zhang

**Affiliations:** a School of Nursing, Tianjin University of Traditional Chinese Medicine, Tianjin, China; b School of Nursing, Tianjin University of Traditional Chinese Medicine, Tianjin, China.

**Keywords:** analytic hierarchy process, education, nursing, postgraduate, quality evaluation

## Abstract

Nursing is an inseparable job with the healthy life of human beings. High-level nursing talents have a greater influence on patients. It is the future trend for schools to train Nursing Professional Degree Postgraduate, and the evaluation of their education quality is the top priority.

To construct the education quality evaluation index system of Nursing Professional Degree Postgraduate and to determine the weight of each indicator.

Firstly, the indicators of the evaluation index system of education quality were identified from the literature review. Meanwhile, the Delphi questionnaire was designed and 13 experts evaluated and rated the indictors who were invited to conduct two rounds of the questionnaire. The weights associated with the factors were determined using the analytic hierarchy process and percentage methods, Finally, we developed the evaluation index system of education quality for a postgraduate nursing professional degree.

The evaluation system consisted of 4 first-level indicators, 17 second-level indicators, and 71 third-level indicators. According to the weights computed by analytic hierarchy process, first-level indicators are ranked as “Input quality” (0.1273), “Process quality” (0.3111), “Output quality” (0.0846), “Development quality” (0.4770). Among the secondary indicators, experts pay the most attention to career development (0.3180). The top three indicators of third-level indicators are workplace (0.2385), matching degree between personal expectations and job opportunities (0.1272), and promotion opportunities (0.0795).

The quality index system of nursing postgraduate education is scientific and reliable, and the weight distribution is reasonable. It is an effective tool for evaluating the quality of nursing graduate education.

## 1 Introduction

In China, with the deepening of the reform of the medical and health system, the demand for high-quality health services is increasing, and higher requirements are put forward for the construction of nursing professionals. Especially after the pneumonia epidemic in COVID-19, people from all walks of life paid more attention to the current situation of the construction of nursing staff in China, which also led to a deeper reflection on high-level nursing education.^[1]^

Nurse, as a profession, plays an extremely important role in maintaining and protecting the health of the world population. According to the report, 59% of health care professionals are nurses, and there are about 28 million nurses in the world, including 19.3 million professional nurses, 6 million assistant professional nurses, and the rest are unclassified.^[2]^ Although the global nursing shortage has declined from 6.6 million estimated in 2016 to around 6 million in 2018, the bottom line is that by 2030, there will be a need for 36 million nurses practicing across the globe to meet the needs of every individual on the planet.

The shortage of nursing human resources is a common phenomenon in China. One of the possible strategies to deal with the shortage of nursing talents is to increase the introduction of high-level graduate students. Blass^[3]^ showed that make the point that postgraduate students are the most important source of future contribution to the development of knowledge. A study concluded that improving nurses’ ability at the master's level might improve patients’ outcomes.^[4]^ Besides, it is reported that master nurses provide better professional nursing.^[4]^ It is more likely to become a transformative force for improving clinical nursing.^[5]^ The higher proportion of nurses educated at the baccalaureate level or higher in a hospital, the lower the mortality and failure-to-rescue for surgical patients.^[6]^ This indicates that master's education can improve patients’ outcomes by cultivating professional and personal attributes, thus realizing the potential of nursing specialization.

In developed countries, most nurses with a master's or doctoral degree are generally nurse anesthetists, nurse midwives, nurse practitioners, and clinical nurse specialists, engaging in specialized health care.^[7,8]^ Postgraduate education in foreign countries started early, developed relatively well in training methods and curriculum, and its quality evaluation system is mature and has its characteristics, among which the evaluation system of postgraduate education in Britain, France, and the United States are the most typical. From the perspective of evaluation subjects, the subjects of education quality evaluation are diversified, which mainly shows that universities, government and society participate in the evaluation of education quality in coordination, among which the United States focuses on social evaluation, Britain focuses on the dual evaluation of government and universities and the active participation of society, and France focuses on government evaluation; In terms of evaluation methods, foreign scholars creatively apply the quality evaluation methods in enterprises to the evaluation of education quality, such as using the concept of service quality, total quality management, Six Sigma management, Koch four evaluation models, etc., enriching and develop the evaluation methods of education quality, providing experience and methods for scholars to study; In the establishment of evaluation indicators, scholars used to comprehensively evaluate the investment, process and results of education, and most of the evaluation objects focused on teachers and teaching processes; At present, foreign scholars pay more attention to students themselves and their learning, and the establishment of evaluation indicators is student-centered, and students’ evaluation is included in evaluation activities. Incorporating students into quality evaluation is conducive to understanding the problems existing in the process of education from the perspective of students, to put forward the corresponding improvement measures.

The development and present situation of graduate education quality evaluation in China: China promulgated the Regulations of the People's Republic of China on Degree in 1980, and gradually established an inspection and evaluation system of degree-granting quality since 1985, and then carried out a series of graduate education quality evaluation activities.^[9]^ For example, the comprehensive evaluation of the running level of graduate schools and the overall level of professional degree authorization points in various disciplines, the selection of excellent dissertations, and the evaluation of administrative examination and approval of key disciplines,^[10–12]^ so the current work belongs to the “government evaluation mode.”^[9]^ Since 1994, the evaluation center of degree and postgraduate education in universities and research institutes in China has been established, and some non-governmental organizations, groups, and even individuals have begun to participate in the comprehensive level evaluation of higher education, and at the same time started to rank the education quality of graduate schools in universities, which indicates that the evaluation of postgraduate education quality in China has entered a “diversified evaluation stage.” Up to now, the evaluation process is still under the responsibility of the government departments, and there are still many defects in the evaluation of postgraduate education quality, such as the evaluation index is cumbersome and complicated; many evaluation items for school hardware and teachers’ conditions; few indicators for graduate students’ ability and so on. In the evaluation process, there are a few quantitative indicators and qualitative indicators out of balance, etc.^[13]^ Therefore, it is extremely urgent to establish a unified and standardized evaluation standard for the quality evaluation index system of nursing research education in China.

The theoretical model adopted in this study is Astin's input-environment-output (I-E-O) model is concerned with the way and process of student ’development.^[14]^ I-E-O model provides a theoretical basis for the study of the quality and effect of university education, in which the input mainly refers to “students’ demographic characteristics,” “family background,” “study, and social experience,” “the environment mainly includes students’ academic experience,’ “project participation,” “peer relationship” and “university cultural experience,” and the output mainly refers to “students’ knowledge,” “ability,” “characteristics,” “values” and “behavior patterns” when they leave university campus. Subsequently, Luo^[15]^ put career development into the evaluation system of students’ development and built an input-process-output-development (IPOD) framework based on the I-E-O model to evaluate the quality of education from four aspects: input quality, process quality, output quality, and development quality. In the I-E-O model, based on the connotation of input, process, and output, the quality of students’ development has been improved, including career development and career satisfaction.

At present, the research mainly focuses on nursing education and its influencing factors of undergraduate and professional nursing degrees. Few studies have explored the evaluation index system of the quality of nursing postgraduate education. There is a notable shortage of literature related to the use of the evaluation index system of the quality of nursing postgraduate education in the context of improving nursing level. Therefore, the purpose of this study is to establish an index system of the quality of nursing master's education based on the IPOD mode, to find out the teaching problems earlier and improve the ability to analyze the quality factors of nursing master's education. Thus, the scientific research ability and clinical adaptability of the nursing master were improved.

## 2 Methods

A Delphi study was conducted with 13 experts from 10 nursing colleges to construct an education quality evaluation system for Nursing Professional Degree Postgraduate.

### 2.1 Survey design

A Delphi survey was used in this research. It involves multiple rounds of consultation by using questionnaires to obtain the collective opinion of experts until a consensus is reached.^[16]^ This evaluation index system is useful for the evaluation and improvement of education quality for graduate students of nursing specialty degrees. The query quantity includes general information of experts, the familiarity of experts with query contents, judgment basis table of experts on query contents, and query table of specific indicators. A 5-point Likert-type scale was used ranging from 1 (non-significant) to 5 (very significant) to mark. Based on expert opinions and statistical standards, the index is deleted and increased. Summarize and illustrate all adjustments, then send the revised Evaluation Index system to experts. Until the opinions of all experts tend to be the same, the Evaluation Index system is confirmed. Out of the two-round Delphi consultation, the invited specialists were required to rate the significance and operability of the index system.

### 2.2 Review evidence and generate initial indicators

To developed the initial indicators, a literature search was performed within English (PubMed, Web of Science) and major Chinese (China National Knowledge Infrastructure, Wanfang, and VIP) databases from their inception date to July 2020. The search was performed using the search terms (“education,” “postgraduate,” “nursing specialty degree,” and “quality”).

Based on massive literature and constructed by IPOD Theoretical framework, we developed the initial draft of the quality index system, including 4 first-level indexes, 20 second-level indexes, and 85 third-level indexes. After constructing the Evaluation Index system Draft, the Delphi method was adopted to adjust the Evaluation Index system Draft and confirm the Evaluation Index system.

### 2.3 Experts selection

When constructing the index system by the Delphi method, the choice of consulting experts plays an important role.^[17]^ To make it more specific and reliable, we have made some selection criteria for experts who participated in our research, including ①More than 15 years of nursing education experience; ②Nursing management or clinical nursing; ③Bachelor degree or above; ④Senior professional title or Sub-senior professional title; ⑤Willingness to participate in this research. As for the number of experts, we invited 13 experts based on the criteria and our resources.

Expert familiarity and judgment are used to understand the authority of the experts. Experts familiarity can be divided into five grades: very familiar, relatively familiar, generally familiar, a little familiar, and unfamiliar and assigned 0.9, 0.7, 0.5, 0.3, and 0.1, respectively.

### 2.4 Statistical Methods

The data were input by Excel and analyzed by SPSS20.0. The general information of experts is expressed by frequency and percentage; the measurement data are expressed by mean and standard deviation; the enthusiasm of experts is expressed by the rate of valid questionnaires (the number of returned questionnaires/the number of total questionnaires∗100%), and the coefficient of experts’ authority (Cr) was determined by the coefficient of experts’ judgment and experts’ familiarity. The degree of concentration of experts’ opinions was expressed by importance assignment mean (significance) and standard deviation. The importance assignment means significance <3.5 is taken as the criterion for deleting indicators. The coordination degree of experts’ opinions was expressed by the coefficient of variation (CV) and Kendall harmony coefficient (W). CV > 0.25 was the criterion for deletion.^[18]^ Yaahp software was used to calculate the weight of each index system of education quality for a graduate student of nursing specialty degree.

As for consistency testing, Satty's^[19]^ consistency index and average random consistency index are always adopted, and the ratio of consistency index. and random consistency index is called consistency ratios (CR). When the order n > 2 when the consistency ratio of the judgment matrix is CR < 0.10, the judgment matrix is considered to have satisfactory consistency; if CR > 0.10, the judgment matrix needs to be adjusted.

### 2.5 Ethical considerations

Before the investigation, ethical approval was acquired from Tianjin University of Traditional Chinese Medicine. Experts who meet the inclusion and exclusion criteria were given the informed consent and invited to attend this study. This verbal consent was recorded in the project document.

## 3 Results

### 3.1 Representative sample of experts

Selecting experts from the same background may leaded to a certain degree of deviation from the research results.^[20]^ The characteristics of the experts who participated in the study were shown in Table 1. In this study, we adopted two rounds of expert consultation, enrolling a total of 13 experts from 7 major cities.

**Table 1:** Main characteristics of the expert in two rounds of the Delphi study.

Characteristics	Experts (n = 13)
Age	M=50.23, SD=7.51
Gender	
Male	1(7.69%)
Female	12(92.31%)
Province	
Beijing	2(15.38%)
Tianjin	5(38.46%)
Fujian	1 (7.69%)
Zhejiang	2(15.38%)
Anhui	1(7.69%)
Guizhou	1(7.69%)
Jiangsu	1(7.69%)
Specialty	
Nursing management	3(23.08%)
Clinical nursing	5(38.46%)
Nursing education	5(38.46%)
Professional title	
Senior professional title	7(53.85%)
Sub-senior professional title	6(46.15%)

### 3.2 Authority degree for expert panel and coordination degree of experts

Cr indicated whether the expert consultation results are scientific and reliable. The expert Cr included the evaluation basis of each index and the familiarity of experts with research. Cr was the arithmetic means of the two terms. In this study, the coefficient of experts’ judgment = 1.023 and Experts’ familiarity = 0.854, so Cr = 0.938. It can be considered that the experts participating in this research have high authority. The degree of coordination of expert opinions was reflected by the CV or Kendall harmony coefficient (W). CV had a negative correlation with the degree of coordination of expert opinions, and W value had a positive correlation with the degree of coordination of experts. In the second round, the assignment CV of the primary and secondary indicators were 0.1016 to 0.1250 and 0.1016 to 0.1300, respectively, and the ω was 0.270 to 0.645. Two rounds of expert opinion consultation were performed, which the coordination degree tended to be increased (all *P* < .001), indicating that the coordination degree of the experts for all the indicators improved with increasing rounds of consultation (Table 2).

**Table 2:** The result of expert opinions’ coordination degree.

	Hierarchical level	Index(n)	Kendall's W	Coefficient of variation (CV)	χ^2^	*р*
Round 1	First-level	4	0.510	0.1066∼0.1585	19.899	<.001
	Second-level	20	0.193	0.1102∼0.1512	45.273	<.001
	Third-level	85	0.202	0.1000∼0.1625	220.174	<.001
Round 2	First-level	4	0.645	0.1016∼0.1250	25.163	<.001
	Second-level	17	0.270	0.1016∼0.1300	56.093	<.001
	Third-level	71	0.365	0.1000∼0.1383	331.707	<.001

### 3.3 Results of the first round of Delphi expert consultation

In Delphi Round I, there were four first-level indicators including Input quality, Process quality, Output quality, and Result quality. There were 20 secondary indicators including students’ quality, learning motivation, average resources, subject conditions, professional curriculum quality, practical teaching quality, learning engagement, satisfaction of teaching and auxiliary department, tutors, curriculum teaching, professional practice, academic activities, postgraduate management, academic achievement, personal evaluation, general skills, clinical ability, harvest perception, career development, and career development self-evaluation. A three-level evaluation index system of 85 items on the education quality of nursing graduate students was formed (Table 3). The indicators that reached consensus in Round I was included in the questionnaire of Round II. Combined with the statistical results and the suggestions of the experts in Delphi Round I, the first-level indicator “quality of results” is changed to “quality of development”. And the second-level indicators “learning engagement,” “satisfaction of teaching and auxiliary department,” “academic achievement” and “harvest perception” were deleted. Lots of experts believed that the second-level indicators “general skills” included “clinical competence,” so the “general skills” were divided into “scientific research competence” and “clinical competence” and discussed separately. The third-level indicators are revised. First of all, the teacher structure and teaching method of a repeated content merger, scientific research project evaluation was more in line with the requirements of academic graduate students in our country so it was deleted. Secondly, divide the “general skills” in one's own ability in detail to indicate the content of the project, such as Computer level, Foreign language proficiency (College English Test, Test of English as a Foreign Language, the International English Language Testing System, GRE, Public English Test System, etc.), Statistical method mastery. Finally, the observation time of career development was set as five years after graduation, so that the evaluation criteria are consistent in the time dimension and reflect development more accurately. In short, 15 three-level indexes were deleted, 9 three-level indexes were added, and 5 three-level indexes were adjusted.

**Table 3:** The first round of evaluation index system.

First-level indicator	Second-level indicator	Third-level indicator
Input quality	students’ quality,	······
	learning motivation,	······
	average resources,	······
	subject conditions,	······
	professional curriculum quality,	······
	practical teaching quality,	······
Process quality	learning engagement,	······
	satisfaction of teaching and auxiliary department,	······
	tutors,	······
	curriculum teaching,	······
	professional practice,	······
	academic activities,	······
	postgraduate management,	······
Output quality	academic achievement,	······
	personal evaluation,	······
	general skills,	······
	clinical ability,	······
	harvest perception,	······
Result quality	career development,	······
	career development self-evaluation.	······

### 3.4 Results of the second round of Delphi expert consultation

After Delphi Round I, there were four first-level indicators, seventeen second-level indicators, and seventy-nine third-level indicators for the index system of education quality for graduate students of nursing specialty degrees. In Delphi Round II, experts had a high concentration on indicators at all levels, indicating that experts tend to agree. So, the correspondence ends. Combined with the data results, eight third-level indicators such as “publishing research results,” “competition winning situation” and “organization and coordination ability” were deleted. Finally, the index system of education quality for graduate students of nursing specialty degrees was formed, which includes four first-level indexes, seventeen second-level indexes, and seventy-one third-level indexes. Also, in Delphi Round II, the weight of indicators was given by experts.

### 3.5 Consensus in opinions from consultant experts

Therefore, experts adopted the judgment matrix to determine the importance value of each indicator. Result showed that the consistency of all index at all levels has been verified by the consistency test with CR < 0.10, demonstrating the logical consistency is satisfying while ensuring scientific and reliability.

### 3.6 Final indicators and their weights

After the two-round Delphi study, four first-level indicators, seventeen second-level indicators, and seventy-one third-level indicators were compiled. The results are shown in Table 4. In the first-class indicators, quality of development (0.4770) was the most important indicator. And process quality (0.3111) is the second most important indicator.

**Table 4:** The weights coefficient of each indicator.

First-level indicator (Weights)	Coefficient of variation	Second-level indicator (Weights)	Coefficient of variation	Third-level indicator (Weights)	Coefficient of variation
Input quality (0.1273)	0.1140	Students’ Quality (0.0195)	0.1204	Educational background (first degree, graduate school, etc.) (0.0167)	0.1354
				Comprehensive quality (political and ideological quality, scientific research quality, etc.) (0.0028)	0.1032
		Learning Motivation (0.0471)	0.1300	Personal interest (0.0046)	0.1032
				Career development needs (0.0157)	0.1161
				Affected by family factors (0.0268)	0.1226
		Average Resources (0.0114)	0.1140	Teacher-student ratio (0.0034)	0.1354
				The average number of health items (0.0054)	0.1383
				Average Health Project Funds (0.0021)	0.1275
				Number of books and databases per student (0.0006)	0.1048
		Subject Conditions (0.0301)	0.1250	Quantity and quality of teach bases (0.0149)	0.1250
				The number of scientific research projects at or above the provincial or ministerial level (0.0059)	0.1182
				Number of academic conferences held at home and abroad (0.0094)	0.1204
		Professional Curriculum Quality (0.0078)	0.1121	Professional course content setting (0.0019)	0.1121
				Teaching idea and method (0.0007)	0.1016
				Structure of teaching staff (0.0019)	0.1121
				Course effect evaluation (0.0034)	0.1140
		Practical Teaching Quality (0.0114)	0.1140	Organizational management (0.0034)	0.1140
				Process Evaluation of Practical Teaching Effect (0.0019)	0.1121
				Terminal evaluation of practical teaching effect (0.0062)	0.1204
Process quality (0.3111)	0.1016	Tutors (0.1038)	0.1204	Teachers’ morality (0.0043)	0.1000
				Teachers’ professional accomplishment (0.0060)	0.1048
				Teachers’ academic qualifications, degree structure (0.0088)	0.1032
				Title Structure of Subject Teachers (0.0088)	0.1048
				The age structure of subject teachers (0.0364)	0.1226
				Professional level of teachers (famous teacher, award-winning teacher, teaching level, etc.) (0.0213)	0.1140
				Funding for teachers (0.0060)	0.1032
				Cultivate students’ moral character (0.0122)	0.1066
		Curriculum Teaching (0.0211)	0.1048	Talent training program (0.0048)	0.1121
				syllabus (0.0025)	0.1066
				Teaching content (0.0032)	0.1083
				Teacher-student interaction (0.0019)	0.1048
				Evaluation of teaching effect (0.0073)	0.1161
				Teaching feedback (0.0014)	0.1032
		Professional Practice (0.0323)	0.1066	Practice plan (0.0054)	0.1016
				Practice content (0.0108)	0.1032
				Practice duration (0.0108)	0.1066
				Practical feedback (0.0054)	0.1016
		Academic Activities (0.0472)	0.1066	Attend academic conferences at home and abroad (0.0059)	0.1032
				Attend academic lectures (0.0059)	0.1032
				Academic reporting (0.0354)	0.1327
		Postgraduate Management (0.1067)	0.1226	Management team (0.0529)	0.1327
				Pay attention to students’ ideological and moral cultivation (0.0351)	0.1250
				Reasonable rules and regulations (0.0111)	0.1066
				Positive feedback (0.0075)	0.1048
Output quality (0.0846)	0.1016	Personal Evaluation (0.0257)	0.1204	Political quality (0.0073)	0.1048
				Moral accomplishment (0.0045)	0.1032
				Critical thinking (0.0045)	0.1032
				Humanistic care and empathy (0.0045)	0.1032
				Autonomous learning ability (0.0025)	0.1016
				Establish an interpersonal relationship and communication skills (0.0025)	0.1016
		General Skills (0.0171)	0.1161	Computer level (0.0084)	0.1102
				Foreign language proficiency (CET, TOEFL, IELTS, GRE, PETS, etc.) (0.0033)	0.1048
				Statistical method mastery (0.0053)	0.1066
		Scientific Research Ability (0.0054)	0.1016	Find nursing professional problems (0.0012)	0.1016
				Literature retrieval reading ability (0.0007)	0.1000
				Scientific research and design ability (0.0012)	0.1016
				Scientific research implementation ability (0.0007)	0.1000
				Ability to transform scientific research achievements (0.0018)	0.1032
		Clinical Ability (0.0364)	0.1226	Professional knowledge level (0.0019)	0.1016
				Clinical professional skills (0.0019)	0.1016
				Nursing evaluation ability (0.0029)	0.1032
				Nursing decision-making ability (0.0029)	0.1032
				Ability to deal with the specialist problem (0.0047)	0.1066
				Clinical teaching ability (0.0068)	0.1083
				Professional consulting ability (0.0068)	0.1083
				Health education ability (0.0038)	0.1048
				Cooperative capacity (0.0047)	0.1066
Development quality (0.4770)	0.1250	Career Development (0.3180)	0.1250	Workplace (eastern, central and western; Taiwan, Hong Kong and Macao regions; Abroad) (0.2385)	0.1354
				Promotion opportunities (0.0795)	0.1250
		Self-evaluation of career development (0.1590)	0.1204	Individual expectation and job matching degree (0.0318)	0.1204
				The matching degree between personal expectations and job opportunities (0.1272)	0.1383

CET = College English Test, IELTS = the International English Language Testing System, PETS = Public English Test System, TOEFL = Test of English as a Foreign Language.

In the secondary indicators, the weight of career development is the highest (0.3180). Among other indicators, self-evaluation of career development (0.1590), postgraduate management (0.1067), and tutors (0.1038) are of greater weight.

In the three-level indicators, they were arranged from high to low according to the weight value. The top seven indicators were provided by the duty station (0.2385), individual expectation and job offer matching degree (0.1272), job promotion (0.0795), the management team (0.0529), age structure of subject teachers (0.0364), academic reporting (0.0354), and paying attention to students’ ideological and moral cultivation (0.0351).

## 4 Discussion

In this study, we chose experts in nursing education, clinical nursing, and nursing management as our consulting objects. We also consider the geographical representation of experts and balanced the differences in the professional background of experts from different educational and cultural fields, to reduce the cognitive impact of regional bias.

The enthusiasm of experts shows the degree of concern for the study, usually reflected by experts putting forward their opinions and the recovery rate of the questionnaire. It is generally accepted that a recovery rate of 50% is the minimum requirement for pooled analysis of expert opinion, with recovery rates of more than 60% indicating relatively high enthusiasm, it exceeding 70% illustrating very high enthusiasm.^[21]^ The corresponding proportions of experts who gave full-score were increasing. The questionnaire recovery rate and expert opinions were ideal in this study, indicating that the experts showed better attention and continuing study enthusiasm with each round of consultation.

The authority of expert consultation is one of the key factors involved in the construction of the index system. Based on previous research, an expert consultation Cr >0.7 was considered to be reliable, and an Cr of 0.8 indicates even higher reliability of the experts’ judgment.^[22]^ In this study, the Cr of the experts is 0.938, which indicated that the experts are very familiar with the research topic and have high authority.

The coordination coefficient reflects the coordination degree of the expert opinion, and it is generally in the range of 0.3 to 0.5.^[23]^ In this study, three rounds of expert consultation revealed a coordination coefficient greater than 0.3, and the coordination degree improved significantly with each round (*P* < .001), indicating that the experts an increasing coordination degree concerning all the indicators.

The theoretical model of IPOD was put forward by Chinese scholars and developed according to the IEO model proposed by American scholar Astin. In terms of index weight, “development quality” is the item with the highest weight among the four first-level indicators. This index is an objective and subjective evaluation of the career development of graduate students after graduation. This result is consistent that Nerad, an American scholar, has done a “10-year survey on doctoral graduation”, which measures the quality of doctoral education according to the subjective evaluation of doctoral students’ educational experience and career development experience.^[24]^ The National Association of Professional Postgraduates in the United States and the Institute of Higher Education in the United Kingdom have also investigated and studied the evaluation of postgraduate education experience and related development quality.^[25]^ After graduation, the job treatment, development prospect, and whether or not to engage in the profession corresponding to the major have become important objective indicators to measure the development quality of graduate students.^[26]^ The training programs of professional degree postgraduates in most domestic universities show that “market demand-oriented” is the professional principle that should be followed in the development of professional degree postgraduate education.^[27]^

Among the secondary indicators, experts pay the most attention to career development. The results of a qualitative interview show that the clinical work of nursing postgraduates is the same as ordinary nurses, without full application and reflection of knowledge and professional value learned in the training stage of professional masters.^[28]^ Some Chinese scholars believe that if the mode and content of postgraduate education can be effectively combined with social needs, it will promote the future career development of postgraduates.^[29]^ Qualitative research on male nurses in Macao shows that nurses’ motivation in nursing work mainly focused on future career development opportunities.^[30]^ A study in the United States found that despite the desire to become a registered nurse, few assistants develop their careers within the health care environment.^[31]^ In a word, the professional development of nurses is the most discussed topic in today's society.

In this study, the rank of third-level indicators is workplace, the matching degree between personal expectations and job opportunities, promotion opportunities, and so on. Compared with nursing undergraduates who prefer to work in clinical nursing in the megacity, graduate students prefer to teach in universities or colleges in small cities.^[32]^ Furthermore, when looking for jobs after graduation, doctoral students may consider their professional relationships, work experience, or achievements of their previous work units. This phenomenon is helpful for nursing institutions in underdeveloped small and medium-sized cities to recruit nursing postgraduates and promote rational allocation of nursing human resources, instead of focusing on first-tier cities.^[33]^ These three indicators are all focused on how to connect nursing graduate education with work. Some studies have shown that it is easier for nursing graduate students to be promoted, and it is also competitive to have a positive attitude towards nursing work. The employment rate of nurses in economically developed areas is higher, and job matching is higher.^[34,35]^ Zamanzadeh^[36]^ reviewed the existing literature on the motivation factors for men to enter the nursing industry. They found that practical factors, such as working conditions, remuneration, and job security, are all related to favorable results. Research shows that due to the lack of positive social status, a large number of students choose to transfer or drop out. Dissatisfaction with nursing is considered to lead to students’ depression and resignation, leading them to leave their major and continue to study.^[37]^ Nursing students from Croatia agree that employment convenience is one of the major advantages. Part-time students added that they have many opportunities to be promoted in their current workplace. In many cases, part-time students in Croatia and Slovenia have been employed as nursing staff in medical and health institutions. Obtaining a diploma provides them with a good opportunity to get a better working environment. As stated in Slovenian and Croatian laws, the nursing team leader can only be a nurse with complete undergraduate or postgraduate nursing education.^[38]^ Vocational education before graduation will help new nurses to adapt to the increasing expectation of professional practice.^[39–41]^ Previous studies have shown that positive changes in salary, autonomy, and responsibilities can improve nurses’ attitude towards the nursing profession and strengthen the implementation of reform.^[42]^ According to the theory of fairness, individuals who think that employers treat and reward their abilities or work experience fairly will be more satisfied, motivated, and dedicated.^[43]^ The mismatch between expectation and reality, as well as the unsatisfied expectation, leads to the newly graduated young nurses leaving their profession.^[44,45]^

## 5 Conclusion

This evaluation index system of education quality for postgraduate nursing professional degree includes four first-level indexes (Input quality, Process quality, Output quality, and Development quality), 17 secondary indexes, and 71 tertiary indexes. Moreover, the expert motivation, expert authority, and expert coordination were promising, indicating the construction of the Evaluation Index System of education quality for the postgraduate nursing professional degree was scientific.

In more detail, it can also be used for teachers and administrators of the education department to make training plans for nursing graduate students. There is no comprehensive evaluation method to evaluate the education quality of nursing graduate students so far because there are always some personal opinions and deviations in evaluating education quality. However, the evaluation index system established in this study combines the advantages of qualitative and quantitative evaluation indexes and then weights them according to their importance in nursing education. It is hoped that the framework will provide a method for evaluating the education quality of nursing graduate students in the future.

## Author contributions

**Conceptualization:** Wentong Wei, Yanhui Liu, Yannan Kang, Ruzhen Luo.

**Data curation:** Wentong Wei, Jingying Liu, Ruzhen Luo.

**Formal analysis:** Wentong Wei, Yannan Kang.

**Investigation:** Wentong Wei.

**Methodology:** Wentong Wei, Jingying Liu, Yanhui Liu.

**Project administration:** Yanhui Liu.

**Writing – original draft:** Wentong Wei, Jingying Liu, Yannan Kang, Xiaohong Zhang.

**Writing – review & editing:** Wentong Wei, Yanhui Liu, Yannan Kang, Ruzhen Luo, Xiaohong Zhang.
